# A blemish on bipolar disorder: aggressive behaviour

**DOI:** 10.1192/j.eurpsy.2022.1043

**Published:** 2022-09-01

**Authors:** R. Zanardi, E. Manfredi, F. Attanasio, M. Carminati, C. Colombo

**Affiliations:** 1 IRCCS San Raffaele Scientific Institute, Psychiatry - Mood Disorders, Milano, Italy; 2 Università Vita-Salute San Raffaele, Psychiatry, Milano, Italy

**Keywords:** aggressiveness, stigma, violence, bipolar disorder

## Abstract

**Introduction:**

Many studies have searched for an association between violence and psychiatric diagnoses, without providing a confirmative result.

**Objectives:**

We have sought to deepen this topic, analysing different aspects of aggressivity, focusing on a specific diagnosis and its particular phases of illness, and looking for a correlation between psychiatric co-diagnoses and outpatients’ visits adherence.

**Methods:**

We studied 151 bipolar type I inpatients presenting complaint, past medical and family history; we collected information about lifetime hetero/self-aggressive behaviours, irritability, agitation, suicide attempts, alcohol, or substance abuse.

**Results:**

**Figure:**
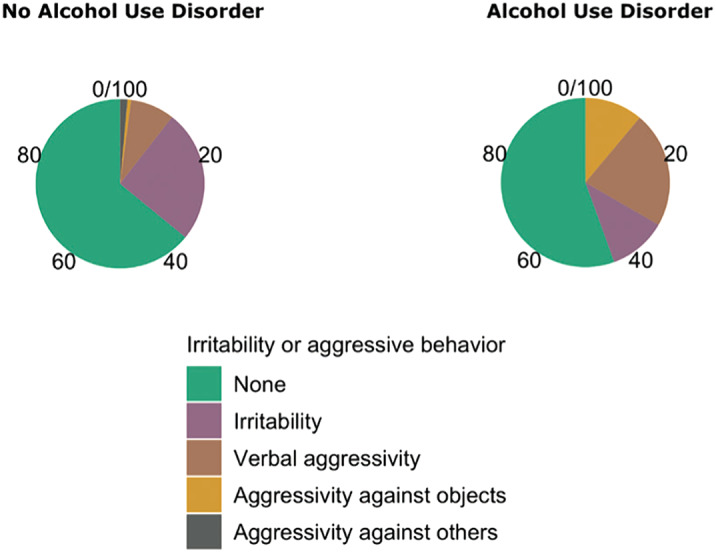

**Figure:**
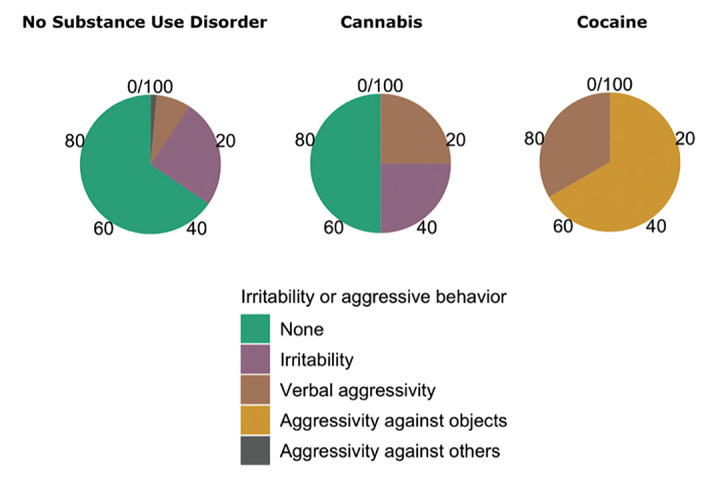

The overall aggressivity in our sample resulted in 11.92% of cases, while the number of aggressive episodes during euthymia decreased to 2.64%, close to the population without psychiatric disorders. Personality disorders and alcohol abuse appeared to be the main risk factors for irritability [Fig. 1]; substance abuse for both irritability and hetero-aggressive behaviour [Fig. 2]. We observed that subjects who displayed better compliance to follow-up visits exhibited a significant lower aggressive behaviour than less adherent subjects. Moreover, our data disconfirm the common conception that correlates the presence of psychotic features to violence.

**Conclusions:**

Studying aggressive in a bipolar population, we observed that the rare episodes of aggressiveness were condensed in active phases of illness and mainly related to alcohol or substance abuse, while violent acts during long periods of wellbeing appear in line with those of the general population. We are confident our data might be helpful in deconstructing the stigma that a psychiatric diagnosis equals to violent behaviour.

**Disclosure:**

No significant relationships.

